# Reward coding across mouse visual cortical areas

**DOI:** 10.3389/fnbeh.2026.1944695

**Published:** 2026-10-28

**Authors:** Johseph P. G. Souza, Anne B. Vieira, Vicente Ventura, Julio C. de Lima, Gabriel Arcanjo, Edson V. de Paula, Matéus Sales, Ana B. A. Souza, Justo E. Alvarez-Jácobo, Antonio J. Fontenele, Leandro A. A. Aguiar, Maíra A. de Santana, Nuno Sousa, Nivaldo A. P. de Vasconcelos

**Affiliations:** 1 Departamento de Engenharia Biomédica, Universidade Federal de Pernambuco, Recife, PE, Brazil; 2 Departamento de Física, Universidade Federal de Pernambuco, Recife, PE, Brazil; 3 Departamento de Psicologia, Universidade Federal de Pernambuco, Recife, PE, Brazil; 4 Departamento de Engenharia Mecânica, Universidade Federal de Pernambuco, Recife, PE, Brazil; 5 Department of Physics, University of Arkansas, Fayetteville, AR, United States; 6 Centro de Informática, Universidade Federal de Pernambuco, Recife, PE, Brazil; 7 Centro Universitário Max Planck (UniMAX), Indaiatuba, SP, Brazil

**Keywords:** cortical hierarchy, mouse, Neuropixels, reward-related suppression, visual cortex

## Abstract

The visual cortex is an established site of reward-related signaling, but this evidence comes almost entirely from the primary visual cortex and from trained associative paradigms, leaving open whether and how a dedicated reward signal is organized across the visual cortical hierarchy as a whole. Here we show, using 103 sessions of the Allen Visual Behavior Neuropixels dataset in which head-fixed mice performed a visual change-detection task, that reward is encoded throughout the mouse visual cortex. Aligning the activity of 54,039 units to reward delivery, we identified reproducible peri-reward responses with a split-half test against an inter-spike-interval null, and retained only units whose modulation was reward-specific across two contrasts: active behavior vs. passive replay, and hit vs. miss. A substantial cohort of units, distributed across all six visual areas, was reward-modulated. This modulation was dominated by suppression, whose predominance decreased systematically along the functional hierarchy, from the primary visual cortex towards higher-order areas, and was concentrated in layer 4 and in narrow-spiking neurons. At the population level, the reward-modulated cohort operated in a lower-variability regime than the surrounding population in every area. Decomposing the peri-reward responses showed that they do not fall into discrete types but form a shared continuum of temporal profiles, common across areas, within which an anticipatory ramp preceding reward was most prevalent in the primary visual cortex. These findings indicate that the mouse visual cortex carries a distributed, predominantly suppressive and anticipatory reward signal, hierarchically graded yet built from a shared temporal repertoire.

## 1 Introduction

Reward processing is among the most fundamental functions of the nervous system: by signaling which outcomes are worth pursuing, it guides learning, motivation, and adaptive decision-making (Schultz, 2000, 2015) throughout the animal kingdom (Barron et al., 2010). Such processing has traditionally been attributed to subcortical dopaminergic circuits, organized in mammals around the mesolimbic system, an evolutionarily ancient and conserved pathway (Costa and Schoenbaum, 2022). Value attribution, however, is not confined to this circuit: hedonic impact, associative learning, and incentive salience are dissociable processes (Berridge and Robinson, 1998). If value processing is distributed, areas classically regarded as purely sensory may take part in it, and the visual cortex is the most studied case.

There, reward-related responses emerge with learning. When a visual cue is paired with a delayed reward, a fraction of primary visual cortex neurons come to encode the interval until its arrival (Shuler and Bear, 2006), a signal whose acquisition depends on cholinergic input from the basal forebrain (Chubykin et al., 2013; Liu et al., 2015). Reward association sharpens the representation of the stimuli that predict it and, in the same cells, gives rise to an anticipatory signal that strengthens as the animal learns (Poort et al., 2015; Goltstein et al., 2018). Reward and self-motion signals become directly accessible in the mouse primary visual cortex (Pakan et al., 2018) and, under the inside-out framework of cortical processing (Buzsáki, 2019), the cortex actively modulates its own sensory channels. The visual cortex is therefore an established site of reward-related signaling, and not a purely sensory relay.

This body of evidence, however, comes almost entirely from the primary visual cortex, from trained associative paradigms, and describes mainly how reward-predicting stimuli come to be represented. What happens across the rest of the visual cortex remains open. The mouse visual cortex is not a single area but a set of interconnected fields organized along a functional hierarchy, with a visual response latency and receptive-field size increasing from the primary visual cortex at the base to the anteromedial area at the top (Siegle et al., 2021). A reward signal could therefore vary systematically along this hierarchy, yet whether it in fact traverses it, what form it takes around reward itself, how it is distributed across the population, and what time course it follows remain unknown.

The last two decades have seen remarkable advances in large-scale electrophysiology, now allowing dense simultaneous recordings of the activity of thousands of neurons across multiple brain areas in behaving animals (Nicolelis et al., 1997; Buzsáki, 2004; Stevenson and Kording, 2011; Urai et al., 2022; Hong and Lieber, 2019). The Allen Visual Behavior Neuropixels public dataset offers the scale to address this, with activity recorded simultaneously across six visual cortical areas while head-fixed mice performed an image change-detection task. Its primary description, however, decomposed this activity into sensory, change, engagement, novelty, and action components, without a reward-coding axis, leaving uncharacterized whether a dedicated reward signal, separable from those variables, is present across the visual cortex (Bennett et al., 2026).

Here, we show that a substantial fraction of the recorded units in the visual cortex are modulated during the reward period by a signal dominated by suppression, whose predominance decreases systematically along the functional visual hierarchy and is most pronounced in narrow-spiking neurons. In its dominant temporal structure, this modulation takes the form of an anticipatory ramp that precedes reward and is specific to it, separable from the response to the visual stimulus.

## 2 Materials and methods

### 2.1 Experimental dataset and behavioral paradigm

We analyzed extracellular recordings from 103 sessions of the Allen Visual Behavior Neuropixels dataset (Bennett et al., 2026), acquired with Neuropixels 1.0 probes in 54 head-fixed trained adult mice performing a visual change-detection task. The dataset is openly available from the DANDI Archive as dandiset 000713 (version 0.240702.1725, https://doi.org/10.48324/dandi.000713/0.240702.1725), and the task and data are documented by the Allen Institute at https://portal.brain-map.org/circuits-behavior/visual-behavior-neuropixels. Recordings targeted six cortical visual areas: the primary visual cortex (VISp) and the lateral (VISl), anterolateral (VISal), posteromedial (VISpm), anteromedial (VISam), and rostrolateral (VISrl) areas, yielding 54,039 units in total.

Each session comprised six stimulus blocks, of which we analyzed two: the active behavior block (block 0), which contained the task, licking, and reward, and the passive replay block (block 5), which repeated the same natural-image sequence with neither task nor reward, as illustrated in Figure 1. We verified this block assignment in all 103 sessions and confirmed that no lick or reward events occurred during passive replay. Reward-aligned analyses used performance-contingent rewards only; automatic priming rewards were excluded.

**Figure 1:**
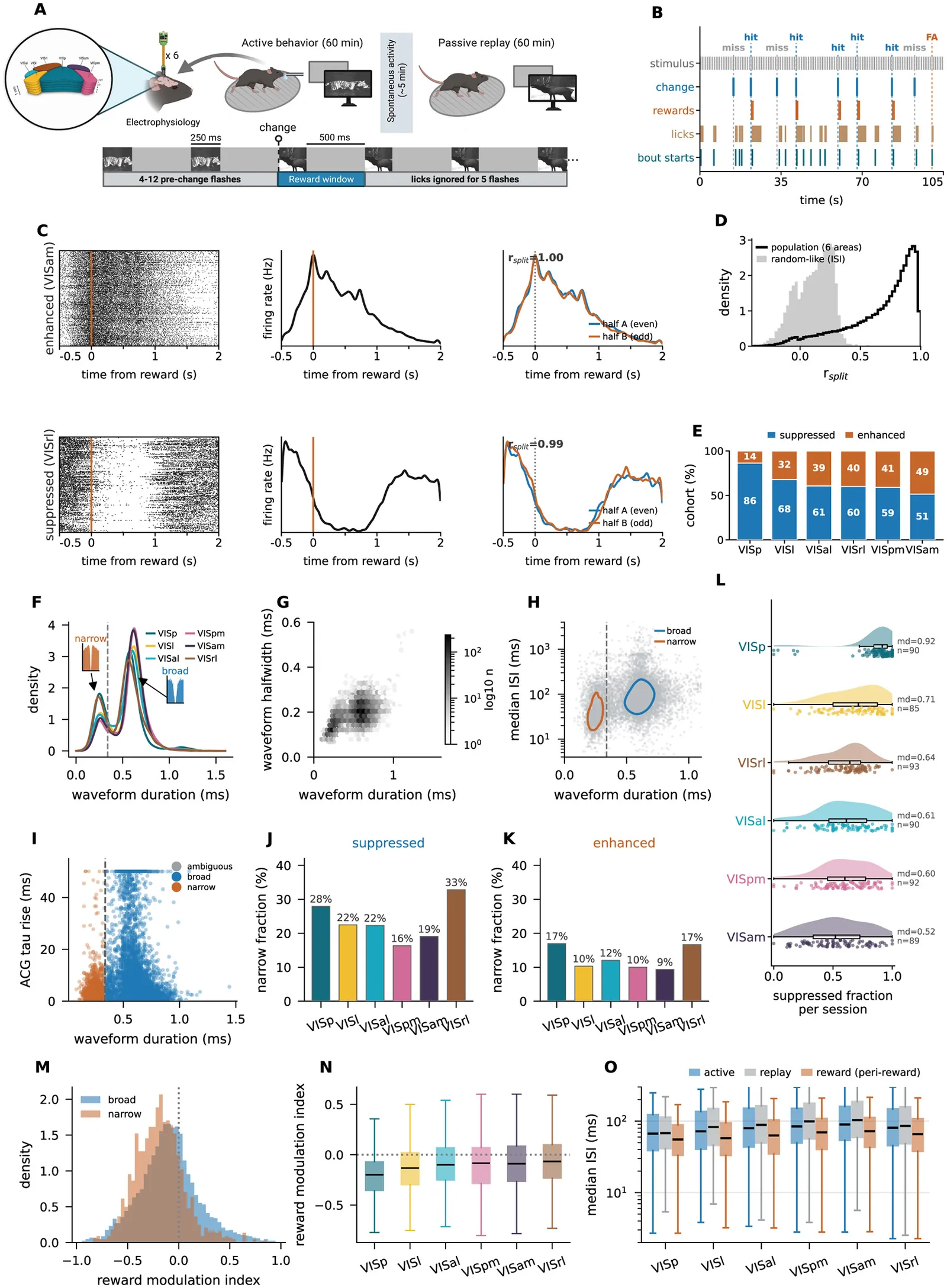
The mouse visual cortex encodes reward: identification, regional distribution, and characterization of reward-modulated neurons. **(A)** Data origin and paradigm. Neuropixels probes (×6) recorded from six visual areas (VISp, VISl, VISal, VISpm, VISam, VISrl) of the Allen Visual Behavior dataset across active-behavior, spontaneous-activity, and passive-replay blocks. Bottom, the change-detection structure: 250 ms image presentations separated by 500 ms gray screens, with water delivered in a response window after an image change (licks ignored for five flashes after reward). **(B)** Behavioral series from one session, temporally aligned: stimulus, image changes, rewards, licks, and bout starts, with hits, misses, and a false alarm (FA) marked. **(C)** Two example reward-modulated cells, one that increases its firing rate at reward (enhanced; VISam) and one that decreases it (suppressed; VISrl). Left to right: reward-aligned spike raster (*t* = 0); firing-rate peri-event time histogram (PSTH); and the split-half test (odd and even trial halves) with its reproducibility coefficient *r*_split_ (1.00 and 0.99). **(D)** Reproducibility across the cohort: distribution of *r*_split_ for the six areas against the ISI-shuffle (“random-like”) null. **(E)** Directional composition by area. Fully stacked bar of the working cohort (bidirectional, ISI null; *N* = 11, 032: 7, 229 suppressed, 3, 803 enhanced), with per-area *N* below. Suppression predominates along a regional gradient, from VISp (86%) to VISam (51%), where the two directions approach parity. **(F)** Waveform-duration density by area, resolving the broad- and narrow-spiking populations (insets: example broad and narrow autocorrelograms); the dashed vertical line marks the narrow/broad boundary estimated by the Gaussian mixture model (see Methods). **(G)** Waveform halfwidth vs. duration for the cohort (hexbin; color, log_10_ count). **(H)** Median ISI vs. waveform duration for the cohort (gray points, all units), with a density contour enclosing 50% of the density mass drawn separately for the narrow- and broad-spiking units (color, waveform class; dashed line, the narrow/broad boundary of **(F)**). The horizontal axis is truncated at 1.1 ms, which places 80 units (0.7%) outside the panel. Median ISI was lower in narrow-spiking (40.9 ms, *n* = 2, 308) than in broad-spiking units (85.0 ms, *n* = 8, 716). **(I)** Excitatory/inhibitory plane (ACG τ_rise_ vs. waveform duration, trough-to-peak) separating broad/RS (putative excitatory, blue), narrow/FS (putative inhibitory, orange), and ambiguous units (gray). **(J, K)** Narrow-spiking fraction by area within the suppressed **(J)** and enhanced **(K)** subpopulations. **(L)** Per-session suppressed fraction by area (raincloud: half-violin density, boxplot, and one dot per session with ≥ 5 units in that area; median and *n* at right), areas ordered by descending median. Treating each session as the unit of analysis, the **(E)** gradient is reproduced, from VISp (0.92) to VISam (0.52). **(M)** Reward modulation index for broad/RS vs. narrow/FS units. **(N)** Reward modulation index by area (negative medians, consistent with the predominance of suppression). **(O)** Median ISI by area in the active, replay, and peri-reward conditions. Cell types are labeled as putative excitatory or inhibitory; the assignment is putative, as some excitatory neurons are also narrow-spiking. The cohort was identified by split-half reproducibility of the reward-aligned PSTH against an ISI-shuffle null; see Methods. Created in BioRender. Souza, J. P. G. (2026) https://BioRender.com/q5rorf2.

In both blocks, natural images were presented for 250 ms and separated by a 500 ms gray interval, with reward delivered only in the active block. On change trials, one image identity was replaced by another: a lick within the response window was scored as a hit and followed by water delivery, whereas a failure to lick was scored as a miss. Catch trials, in which no image change occurred, were scored as false alarms or correct rejections according to the animal's response. Figure 1B illustrates the behavioral sequence from one session.

### 2.2 Reward-aligned peri-event responses

For each unit, spikes from the active block were aligned to the performance reward delivery in a window from −0.5 to +2.0 s and counted in 10 ms bins. Reward events were retained only when the entire analysis window fell within the active block. Consecutive rewards were separated by at least 6.96 s in this dataset; therefore, no peri-reward window contained a neighboring reward event. Firing rate curves were obtained by dividing the bin counts by the number of events and bin duration. Unless otherwise specified, temporal curves were convolved with a normalised Gaussian kernel with σ = 25 ms. A sensitivity analysis was performed by repeating the reliability calculation without smoothing and with σ ∈ {50, 100, 250} ms.

### 2.3 Split-half detection of reproducible responses

Reward-locked temporal reproducibility was measured directly from the peri-stimulus time histogram (PSTH), without assuming any particular response shape. Within each session, chronologically ordered reward events were divided into interleaved odd and even subsets. A peri-reward histogram was computed for each subset, and split-half reliability was defined as the Pearson correlation between the two histograms across time bins,


$$\begin{array}{l} r_{\text{split}}=\text{corr}\left({\text{PSTH}}_{\text{odd}},{\text{PSTH}}_{\text{even}}\right). \end{array}$$ (1)

Interleaving distributed slow within-session drift across the two subsets. The primary analysis used the default resolution defined above, at which the separation between observed and null reliability was maximal in the prespecified resolution sweep.

Significance was assessed by permutation because adjacent PSTH bins are temporally dependent. The primary null was an inter-spike-interval (ISI) shuffle. For each unit, the observed ISIs were randomly permuted and the spike train was reconstructed, preserving spike count and the empirical ISI distribution while disrupting the temporal ordering of spikes relative to reward. Both half-PSTHs and *r*_split_ were recomputed for each of 1,000 permutations. A one-sided permutation probability was calculated as


$$\begin{array}{l} p_{\text{split}}=\frac{1+\sum_{b=1}^{N_{\text{perm}}}1\left(r_{\text{null}}^{\left(b\right)}\geq r_{\text{split}}\right)}{N_{\text{perm}}+1}. \end{array}$$ (2)

The analysis was repeated with a *circular-shift null*, in which the complete spike train was shifted by a random offset and wrapped within the active block. This alternative preserves autocorrelation, bursting, and slow drift while disrupting alignment to reward. The ISI null defined the working cohort, and the circular-shift analysis was retained as a sensitivity analysis.

Permutation probabilities were pooled across sessions and corrected separately within each visual area using the Benjamini–Hochberg procedure at *q* = 0.05. Units passing this criterion were considered to have a reproducible peri-reward response. This detection step identified event-locked reproducibility, but did not by itself distinguish reward from visual, motor, or task-related activity. Reward specificity was therefore evaluated in a separate cross-condition stage.

### 2.4 Cross-condition tests of reward specificity

Cross-condition analyses were restricted to split-half-positive units and used two complementary families of comparisons. For each trial and condition, the response amplitude was the mean firing rate in the interval from 0 to 0.75 s after the response event minus the mean firing rate in the 0.5 s baseline preceding the image change associated with that trial. Anchoring the baseline to the image change, rather than to reward delivery, prevented variability in change-to-reward latency from moving the visual transient into the baseline. Across sessions, the median change-to-reward latency was 0.467 s. Because this single session-level value is used only to position the response window in passive replay, we quantified its within-session dispersion over the 16,330 hit trials: the median within-session standard deviation was 0.104 s (range 0.046–0.174 s), and 3.8% of hits departed from their session median by more than 250 ms. Trial-level latencies cannot be substituted in passive replay, where no reward is delivered, and therefore no such latency exists. As a control for this dispersion, we repeated Family A twenty times, drawing each pseudo-reward latency at random, with replacement, from the empirical within-session distribution of hit latencies instead of using the session median. The contrast was insensitive to the resulting jitter: across the twenty realizations, 99.2% of the cohort continued to pass Family A with an unchanged sign of the specificity index (range 99.1–99.3%) and the index correlated with its original value at ρ = 0.996. The 0 to 0.75 s post-response window was set to the scale of the task's response window, in which a lick within 150–750 ms of an image change earned reward, so that it spanned the immediate consummatory period rather than later, slower components.

Family A compared the active and passive blocks. The active response was aligned to the reward delivered on hit trials. For passive replay, a pseudo-reward time was placed after each replayed image change using that session's median change-to-reward latency. The two trial-level amplitude distributions were compared with a two-sided Mann–Whitney *U* test. Family B provided a within-active-block comparison: the reward-aligned amplitude on hit trials was compared with the amplitude at the corresponding pseudo-reward time after image changes on miss trials. Thus, Family A tested whether the response persisted when the behavioral context and reward were absent, whereas Family B tested whether it persisted after an unrewarded image change within the active task.

Before each family-level comparison, units were required to respond in at least one of the two conditions. Responsiveness was tested with a two-sided Wilcoxon signed-rank test of the per-trial amplitude against zero at *p* < 0.05. This filter prevents nearly zero amplitudes from producing unstable normalized indices. Family-level Mann–Whitney probabilities were corrected with Benjamini–Hochberg false-discovery-rate control at *q* = 0.05, separately for each area and family. Direction was summarized by a specificity index (SI),


$$\begin{array}{l} \text{SI}=\frac{A_{1}-A_{2}}{\left|A_{1}|+|A_{2}\right|}, \end{array}$$ (3)

where *A*_1_ and *A*_2_ are the mean trial amplitudes in the two conditions. The SI captured only the direction and consistency of the cross-condition contrast used to define the cohort; it is distinct from the reward modulation index used below to quantify the amplitude of the peri-reward response (Section 2.5).

The final bidirectional cohort comprised units that passed the ISI split-half gate, were significant after false-discovery-rate correction in both Family A and Family B, and had concordant SI signs in the two comparisons. Units with negative indices in both families were labeled *reward-suppressed*, and units with positive indices in both were labeled *reward-enhanced*. Direction was therefore a property of an accepted unit rather than an inclusion preference. Of 11,271 units significant in both families, 239 had discordant signs (opposite SI sign in Family A vs. Family B) and were excluded, leaving 11,032 units in the working cohort.

### 2.5 Electrophysiological and anatomical characterisation

For each unit, we computed the extracellular waveform trough-to-peak duration and waveform half-width from its mean spike waveform, its mean firing rate over the whole recording, and its anatomical location by querying the Allen Mouse Brain Common Coordinate Framework (CCFv3; Wang et al., 2020). The trough-to-peak duration was the interval from the waveform trough to the following positive peak, and the half-width was the width of the trough measured at half of its minimum amplitude. Putative waveform class was estimated from the trough-to-peak waveform duration of the full visual-area population. A two-component Gaussian mixture model with full covariance and eight initialisations was fitted to this one-dimensional measure. We used a data-driven mixture rather than a fixed literature threshold because the trough-to-peak distribution and the position of its narrow/broad boundary depend on probe geometry and spike sorting, so a per-dataset fit adapts the split to the present recordings. The component with the shorter mean duration was labeled narrow-spiking, and the other broad-spiking. Units with a posterior narrow-component probability from 0.4 to 0.6 were labeled ambiguous and excluded from narrow-versus-broad comparisons; this removed 849 of 53,806 units (1.6%), of which 227 belonged to the cohort (2.1% of it). Widening the ambiguous band to 0.3–0.7 removed 1,280 units (2.4%) and left the results unchanged: the suppressed fraction was 0.790 in narrow and 0.617 in broad units, against 0.790 and 0.619 with the narrower band, and the narrow fraction per area moved by less than 0.004. These labels are electrophysiological classes and were not treated as molecular cell identities.

The amplitude and direction of each unit's peri-reward response were summarized by a reward modulation index *M*, computed on the reward-aligned firing-rate histogram of the active block,


$$\begin{array}{l} M=\frac{{\bar{r}}_{\text{post}}-{\bar{r}}_{\text{pre}}}{{\bar{r}}_{\text{post}}+{\bar{r}}_{\text{pre}}}, \end{array}$$ (4)

where ${\bar{r}}_{\text{post}}$ and ${\bar{r}}_{\text{pre}}$ are the mean firing rates in the post-reward window (0 to +2.0 s) and the pre-reward baseline (−0.5 to 0 s), respectively. Negative values indicate lower firing after reward than in the pre-reward baseline, and positive values, the converse. This index quantifies the amplitude of peri-reward modulation reported by area and waveform class, and is distinct from the cross-condition specificity index defined above.

The pre-reward baseline is deliberately adjacent to reward delivery, following the usual convention for peri-event indices, in which the reference is the state immediately preceding the event. This window overlaps the interval in which the anticipatory archetypes (Section 3.4) peak, so a unit carrying a pre-reward ramp contributes a raised ${\bar{r}}_{\text{pre}}$ and a correspondingly negative *M*. We therefore recomputed *M* for all 11, 032 cohort units with the response window held fixed and three earlier baselines (−1.0 to −0.5, −1.5 to −1.0, and −2.0 to −1.5 s). Retreating the baseline to −1.5 to −1.0 s raises the median *M* from −0.1323 to −0.0351 and lowers the fraction of units with *M* < 0 from 71.6% to 54.5%, with the median changing sign in VISal, VISpm and VISam; the ordering across areas is preserved, VISp being the most negative under every baseline tested. No baseline reaches the preceding reward, the minimum inter-reward interval being 6.96 s, and none discards events at the start of the active block. Two consequences follow for the reading of *M*. First, its magnitude reflects the anticipatory ramp as well as any post-reward change, and this index does not separate them. Second, *M* and the direction label are not equivalent: 44.6% of the units labeled reward-enhanced by the specificity index have a negative *M* under this baseline, against 1.9% under the retreated one. The direction label used throughout, and the regional gradient of Figure 1E, derive from the specificity index and not from *M*, which is reported here as a measure of amplitude only.

Cortical layers were assigned by querying the Allen CCFv3 25-μm annotation volume at the CCF coordinates of each unit, and mapping the resulting structure identifier to its laminar acronym. Layers 1 and 2/3 were grouped as supragranular, layer 4 as granular, and layers 5, 6a, and 6b as infragranular. Layer assignments were checked against the monotonic ordering of both CCF dorsoventral position and position along the probe. Analyses were also repeated after requiring agreement between the CCF-derived area and the area label in the unit table. A layer was assigned to 87.5% of the 54,039 tested units and to 84.9% of the cohort, ranging from 96.6% in VISam to 48.0% in VISrl, and the CCF-derived area agreed with the unit-table label for 63.5% of tested units. To bound the sensitivity of the assignment to registration error, we measured for each unit the smallest displacement along the three CCF axes that changed its laminar label. This quantity is a lower bound on the distance to the nearest layer boundary and assumes nothing about the local orientation of the cortex; laminar comparisons were repeated after restricting to units at least 50 or 75 μm from a boundary.

Regional, laminar, and waveform-class differences in response direction were first summarized as unit fractions. To reduce pseudoreplication, confirmatory comparisons used the recording session as the unit of analysis. Within each session and stratum, the fraction of reward-suppressed units was computed only when at least five eligible units were available. Paired comparisons between two strata used the Wilcoxon signed-rank test; comparisons across the six areas used the Kruskal–Wallis test.

### 2.6 Population spiking variability

Population variability was evaluated across the entire active block of each session based on the coefficient of variation of the summed population activity (Renart et al., 2010; de Vasconcelos et al., 2017; de Paula et al., 2026). For each session and area, units were divided into the final reward-modulated cohort (RW+) and all remaining units (RW-), and their spikes were summed into a single population count in non-overlapping 50 ms bins,


$$\begin{array}{l} n\left(t\right)=\underset{j\in \text{group}}{\sum }\underset{k}{\sum }1\left[t\leq t_{j}^{k}<t+\Delta T\right],\;\Delta T=50\text{ ms}, \end{array}$$ (5)

where $t_{j}^{k}$ denotes the *k*-th spike time of unit *j*. Each active block was partitioned into non-overlapping 10 s windows, and within each window *i* the coefficient of variation was computed as


$$\begin{array}{l} {\text{CV}}_{i}=\frac{\text{SD}\left(n\right)}{\text{E}\left(n\right)}, \end{array}$$ (6)

where E(*n*) is the mean spike count and SD(*n*) the standard deviation of the spike count, both taken over the 50 ms bin counts within window *i*.

Units with a mean firing rate below 0.2 Hz over the whole recording were excluded from all groups as a quality-control step. Because the variability of a summed population depends on population size, RW- and the complete population were repeatedly sampled without replacement (20 draws, averaged) to match the number of RW+ units. Curves were aligned to active-block onset and averaged across sessions. For the scalar comparison reported in the Results, the median CV value across time was calculated for each session, and RW+ and RW- were compared within the area using a paired Wilcoxon signed-rank test.

Because the coefficient of variation depends on the mean firing rate as well as on the number of units, the comparison was repeated with the rate distribution matched between groups in addition to the unit count. Within each session and area, whole-recording firing rates were binned into octiles of log_10_ rate over the union of RW+ and RW-, and the same number of units was drawn from each group within every bin, so that the two subsamples share both size and rate histogram by construction; 20 draws were averaged, as for the count-matched version. The pairwise correlations between the CV(t) series were additionally tested against a null that preserves the autocorrelation of each series and destroys only the relation between series, by shifting each series circularly by an independent random offset and, in a second variant, by permuting blocks of 30 consecutive windows (1,000 permutations each).

### 2.7 Unsupervised decomposition of temporal response profiles

For each unit in the final cohort, a reward-aligned PSTH was constructed from −0.5 to +1.5 s in 10 ms bins, smoothed with a 25 ms Gaussian kernel, and standardized within a unit. A 100 ms padding interval was included before smoothing and removed afterwards to reduce edge artifacts. These standardized profiles formed an 11, 032 × 200 response matrix.

To ask whether these responses are better described as discrete response types or as a continuum of mixed profiles, each signed response was represented by two non-negative channels, [max(*X*, 0), max(−*X*, 0)], and decomposed by non-negative matrix factorization (NMF) for ranks 2–8 (Lee and Seung, 1999, 2000; Fogel et al., 2016); we refer to the resulting components as archetypes. The signed form of each archetype was recovered by subtracting its negative channel from its positive channel. NMF used coordinate descent, a tolerance of 10^−4^, and a maximum of 600 iterations. Archetype stability was measured with five repeated random fits compared with a fixed reference fit after optimal cosine-similarity matching. Variance explained and a cross-fit stability measure, in which disjoint random halves of the units were factorized independently and their archetypes matched by cosine similarity, are reported for every rank from 2 to 8 in Supplementary Figure S1. For the six-archetype representation, each unit's effective number of archetypes was defined as the exponential of the entropy of its normalized NMF weights. To contrast the continuous description with a discrete one, the NMF reconstruction was compared, using variance explained in the original signed response space, with a hard single-archetype assignment (a full-covariance Gaussian mixture fitted after principal component analysis) and with a rank-matched principal-component reconstruction.

### 2.8 Statistical analysis

All tests were two-sided except the one-sided permutation test for positive split-half reliability. False-discovery-rate control used the Benjamini–Hochberg procedure at *q* = 0.05 within each prespecified family of area-wise comparisons. Nonparametric tests were used for trial-level and session-level comparisons because the analyzed quantities were bounded, skewed, or both. Exact sample sizes and the relevant unit of analysis are reported with each result. Analyses were performed in Python 3.11 using numpy, scipy, pandas, and scikit-learn. Randomized procedures used fixed seeds defined in the production scripts.

## 3 Results

### 3.1 Reward coding across the visual cortex

To determine whether the mouse visual cortex encodes reward and which neurons carry this signal, we aligned each unit's spikes to reward delivery in the active block and identified reproducible peri-reward responses with a split-half test: the correlation between the peri-reward histograms of interleaved halves of the reward events, evaluated against an inter-spike-interval (ISI) shuffle null and corrected within each area (Benjamini–Hochberg, *q* = 0.05). Two example cells illustrate the two directions of modulation, one that increases its firing at reward (VISam) and one that decreases it (VISrl), both with near-ceiling reproducibility (*r*_split_ ≈ 1.0) well above the null, which the full cohort exceeds (Figures 1C, D). Because this detection measures event-locked reproducibility, but does not by itself separate reward from visual or motor activity, we restricted the cohort to units whose modulation was reward-specific in two cross-condition contrasts, active vs. passive replay and hit vs. miss (see Materials and Methods).

We found that reward modulation was widespread across all six visual areas and followed a regional gradient in its sign (Figure 1E). Each unit in the cohort was labeled as reward-suppressed or reward-enhanced according to the sign of its specificity index (SI), required to be concordant across both contrasts. No threshold was applied to *r*_split_ itself. Units entered the cohort by first passing the split-half detection gate, in which the permutation probability of *r*_split_ against the ISI-shuffle null was corrected for false discovery rate, and then being significant after false-discovery-rate correction in both cross-condition families with concordant SI signs (Materials and Methods). The direction label, therefore, followed from the sign of the SI rather than from the magnitude of *r*_split_. This cohort comprised 11,032 units out of the 54,039 recorded across the six visual areas (20.4%; 32.9% of the 33,502 units testable in both contrasts).

Reward-suppressed units (7,229; 65.5% of the cohort) outnumbered reward-enhanced units (3,803; 34.5%) overall, but their relative prevalence varied across areas, being highest in the primary visual cortex and lowest in the anteromedial area. Ordered along the functional visual hierarchy, the per-unit suppressed fraction was 86% in VISp (*n* = 2, 374), 68% in VISl (*n* = 1, 782), 60% in VISrl (*n* = 1, 894), 61% in VISal (*n* = 1, 511), 59% in VISpm (*n* = 1, 765) and 51% in VISam (*n* = 1, 706); the two mid-hierarchy areas, VISrl and VISal, were within one percentage point and not reliably ordered. Correspondingly, the reward-enhanced fraction rose from 14% in VISp to 49% in VISam, where the two signs were nearly balanced. This decline was not an artifact of pseudoreplication: with the recording session as the unit of analysis, the per-session median suppressed fraction fell from 0.92 in VISp to 0.52 in VISam, and the areas differed (Kruskal–Wallis, *p* < 0.001; Figure 1L). The increase in the enhanced fraction from primary towards higher-order visual areas is consistent with a hierarchical organization of reward-related signals, but the alignment with anatomical hierarchy scores (e.g.Hunt and Hayden, 2017) remains to be tested explicitly.

### 3.2 Organisation of reward-modulated cells by waveform, layer, and area

Distinguishing cortical cell types from extracellular recordings has a long methodological history. Barthó et al. (2004) identified putative excitatory (pyramidal) and inhibitory (GABAergic) neurons in the rat cortex from their monosynaptic cross-correlogram interactions and found that, among several candidate features, the duration of the wideband spike waveform gave the most reliable separation of the two populations: short-duration, narrow spikes marked putative fast-spiking interneurons, whereas longer, broader spikes marked putative pyramidal cells. This waveform-based dichotomy has since been extended and applied at scale. Unsupervised clustering of spike shapes dissociates several functionally distinct classes across primate cortical areas (Trainito et al., 2019), and the same regular-spiking/fast-spiking (broad/narrow) division is now standard for large-scale mouse Neuropixels surveys, including the Allen visual-system dataset used here (Siegle et al., 2021); in visual cortex it has been used to relate reward-related activity to inhibitory circuits (Monk et al., 2020). Adopting this framework, we examined how the reward-modulated cohort is distributed along the waveform-duration axis (Figure 1F).

Along this axis, the cohort separated into the two expected populations, narrow-spiking (putative fast-spiking, inhibitory) and broad-spiking (putative regular-spiking, excitatory), a separation that also held in complementary metrics of waveform shape (half-width) and of spiking statistics (median inter-spike interval and autocorrelogram rise time; Figures 1G–I, O), providing a check independent of spike duration alone. The narrow fraction was highest in VISp and VISrl (approximately 26%) and lower in the remaining areas (14–19%; Figures 1J, K), and the reward modulation index (*M*) had a negative median in all six areas (Figures 1M, N), consistent with the predominance of suppression. Two asymmetries organized this suppression reproducibly across sessions. First, among units with an assigned cortical layer (from the CCFv3 annotation volume), suppression was concentrated in layer 4: the per-session fraction of suppressed units was higher in this layer than in the others in 70 of 87 sessions (80%), by a median of +0.116 (interquartile range +0.04 to +0.20; paired Wilcoxon signed-rank test, *p* < 0.001). This concentration did not follow from the composition, sampling, or registration of layer 4. Narrow-spiking units were not over-represented there (14.4% of tested layer-4 units, against 20.7% in layer 6a and 15.5% in layer 2/3), and the layer-4 excess persisted within each waveform class (+0.137 in broad- and +0.114 in narrow-spiking units, *p* < 10^−6^ in both), so layer and waveform are two axes rather than one restated. Layer 4 was not over-sampled relative to its contribution to the cohort (18.8% of tested units with an assigned layer against 16.6% of the cohort), and its split-half detection rate matched that of the remaining layers (86.3% against 86.2%), so the effect is not a difference in detection power. It also survived matching the firing-rate distribution across layers (+0.178, against +0.148 unmatched) and restricting to units at least 50 μm from a laminar boundary (+0.176, in 63 of 72 sessions). Second, narrow-spiking units were more frequently suppressed than broad-spiking units, and this difference persisted within each layer (85% of sessions; *p* < 0.001). Both the layer-4 location and the waveform class thus structure reward suppression over the same regional gradient as Figure 1E. We take up the interpretation of these patterns in circuit terms, and the putative nature of the waveform classes, in the Discussion.

### 3.3 Population spiking dynamics of the reward-modulated cohort

The variability of cortical spiking, and not its mean level alone, has long been read as informative about circuit organization: from the observation that the discharge of single cortical neurons is highly variable and that this variability constrains how signals are pooled across an ensemble (Shadlen and Newsome, 1998), to network accounts in which balanced excitation and inhibition set the level of correlated variability (Renart et al., 2010) and structured connectivity generates slow, state-dependent fluctuations of population activity (Litwin-Kumar and Doiron, 2012; Doiron et al., 2016). This line of work has been developed almost entirely around spontaneous and stimulus-evoked activity in sensory cortex, and has rarely been brought to bear on reward coding. Following the same emphasis on discharge variability (Shadlen and Newsome, 1998), but at the level of the summed activity of a functionally defined cohort, we asked whether reward modulation leaves a signature in the variability of the population and not only in the mean response of individual units.

We compared the variability of the summed population firing rate between the reward-modulated cohort (RW+) and the remaining units of the same session and area (RW-). We measured the coefficient of variation (CV) of the summed spike count in a sliding window across the entire active block, with the number of units matched by subsampling, because the CV depends on population size, and a firing-rate filter over the whole recording (0.2 Hz) applied to all groups, which removes units that fail quality control. RW+ operated at a lower variability level than RW- in all six areas (Figures 2A–C; median CV 0.59 in RW+ vs. 0.79 in RW-; paired Wilcoxon signed-rank test per session, median difference between −0.14 and −0.23 across areas, *p* < 0.001 in all), a nearly categorical separation across sessions (rank-biserial ≈−1).

**Figure 2:**
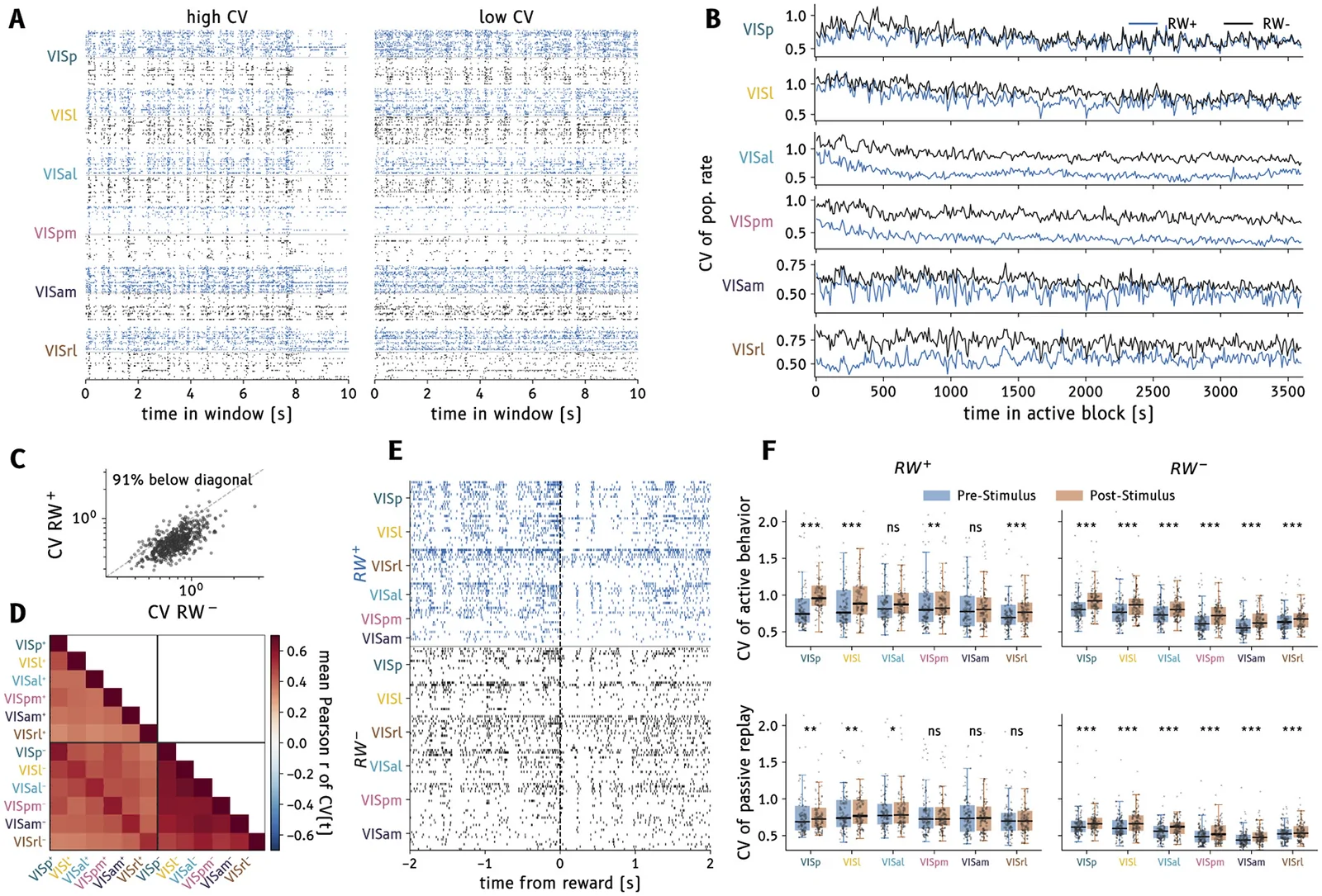
Population variability dynamics of the reward-modulated cohort. Coefficient of variation (CV) of the summed population firing rate in the active block, comparing the reward-modulated cohort (RW+) with the remaining units (RW-) of each session and area, with unit counts matched by subsampling and a 0.2 Hz whole-recording firing-rate filter (see Methods). **(A)** Pooled spike rasters by area (RW+ in blue, RW- in black), contrasting a high-CV and a low-CV window of the summed population activity. For legibility, each area shows an illustrative, equal-sized sample of its more active units (20 Hz firing-rate cap, uniform count per area); the panel illustrates the variability regimes and is not a quantification. **(B)** CV time course by area for a representative session, for RW+ and RW-. **(C)** Per-session, per-area CV of RW+ vs. RW- (log–log; each point is one session × area), with most points below the identity line. **(D)** Pairwise correlation matrix of the CV(t) series across the twelve area × reward-condition combinations (six areas × {RW+, RW-}); color, mean Pearson *r*. All 66 off-diagonal correlations were significant after false-discovery-rate correction (largest $p_{\text{FDR}}=1.0\times 10^{-12}$); the diagonal is 1 by definition. **(E)** Reward-aligned spike raster for an example rewarded (hit) trial (±2 s; dashed line, reward delivery at *t* = 0), contrasting reward-modulated units (RW+, top, blue) with non-modulated units (RW-, bottom, black), sampled across areas and ordered by the functional visual hierarchy (VISp → VISam). For a single modulation sign, RW+ shows the reward-suppressed subset of the cohort, whose firing falls after reward, whereas RW- units show no reward-locked change; this is the single-trial counterpart of the population contrast in **(F)**. **(F)** Pre- vs. post-stimulus CV for RW+/RW- in the active and replay blocks (per session; ***p < 0.001, **p < 0.01, *p < 0.05, ns, not significant; paired Wilcoxon signed-rank test).

The reward-modulated cohort is, however, also a faster-firing one, which the coefficient of variation is sensitive to: over the whole recording, median rates were 5.18 Hz in RW+ against 3.08 Hz in RW-, a ratio of 1.5 to 1.9 across the six areas. We therefore repeated the comparison with the firing-rate distribution matched between the two groups as well as the unit count. The lower-variability regime of RW+ survived in five of the six areas (median difference between −0.02 and −0.12, *p* < 0.01 in all five; 69% of sessions), the exception being VISrl (*p* = 0.07), but attenuated substantially: the pooled median difference fell from −0.19 to −0.06 and the paired rank-biserial correlation from −0.81 to −0.38. Some 67% of the effect measured with unit-count matching alone is therefore attributable to the rate difference between the two groups, and the separation is not categorical once rate is controlled. Matching remains necessary in the other direction as well: without matching the unit count, the sign of the comparison reverses, because the variability of a summed population falls with population size, and RW+ is always the smaller group.

The temporal variability of the CV, moreover, was neither independent across areas nor between the two populations (de Vasconcelos et al., 2017). Correlating the CV(t) series of the twelve combinations of area and reward condition pairwise (Figure 2D), the shared temporal co-fluctuation was strongest within the RW- population (mean cross-area correlation +0.58), intermediate for the cross RW+/RW- block (+0.45), and weakest within the RW+ population (+0.38). All 66 pairwise correlations exceeded a null that preserves the autocorrelation of each series and destroys only the relation between series, obtained by independent circular shifts and by permutation of 300 s blocks (mean null correlation ≤ 0.014 in both variants; *z* ≥ 15 for every pair). The interpretation below, however, rests on the difference between blocks rather than on any pair differing from zero: measured within each session, the excess of the RW- block over the RW+ block had a median of +0.21 and held in 87% of sessions (*p* < 10^−13^). Because a shared slow component enters both blocks of the same session, it cancels in this paired difference. That is, the RW- population shares more common temporal variability across areas than the RW+ population. One possible reading is that the RW- population carries more of a globally shared signal over the session, plausibly linked to state (running, arousal), whereas the reward-modulated cohort is more area-specific and operates in a lower-variability regime. This result is exploratory and carries caveats: the CV does not correct for the mean firing rate, matching the unit count does not remove the sensitivity of the correlation to the noise level of the summed series, and RW+ is a population selected by reward modulation (Figure 1), so part of the contrast may reflect that selection or the temporal structure of the task in the active block rather than an independent synchronization property.

To ask whether this organization of variability is also expressed in alignment with the block's events and whether it depends on the active context, we compared the CV of the summed population firing rate in pre- and post-stimulus windows (image change), separately in the active block and in passive replay, taking the per-session median as the unit of analysis (Figure 2F). In both populations, the CV increased from the pre- to the post-stimulus window, but with unequal robustness. In the non-modulated population (RW-), the post-stimulus increase was significant in all six areas and in both blocks (paired Wilcoxon signed-rank test per session, *p* < 0.001 in all). In the reward-modulated cohort (RW+), the same increase was weaker and area-dependent: in the active block, it reached significance in four of the six areas (VISp, VISl, VISpm and VISrl) but not in VISal or VISam, whereas in passive replay, it was confined to VISp, VISl and VISal, disappearing in the three higher-order areas (VISpm, VISam and VISrl). In VISp, for instance, the median RW+ CV rose from 0.74 (pre) to 0.95 (post) in the active block, against 0.69 to 0.73 in passive replay. A reward-aligned spike raster of example units contrasts the two populations directly at the single-trial level (Figure 2E): reward-modulated units (RW+) reduce their firing just after reward, whereas non-modulated units (RW-) show no reward-locked change, across areas ordered by the visual hierarchy. One possible interpretation is that the increase in the variability of the summed rate after the stimulus is a general cortical response, robust in the non-modulated population, whereas in the reward-modulated cohort, this pre/post modulation is weaker and, in the higher-order areas, contingent on the active context, being reduced in passive replay.

### 3.4 A shared continuum of temporal reward-response profiles

To characterize the temporal structure of the reward response, we decomposed the standardized peri-reward profiles of the cohort (an 11, 032 × 200 response matrix) by non-negative matrix factorisation into six signed archetypes (see Materials and Methods). Ordered by peak latency, these archetypes formed an interpretable repertoire (Figure 3A): three were anticipatory, rising before reward delivery with peaks between −445 and −175 ms, and three were not, comprising a broad, slow archetype, one peaking near reward delivery (−45 ms), and one peaking late in the window (+1405 ms). Most units, however, did not belong to any single archetype but combined several of them. The effective number of archetypes per unit had a median of 2.72, and only 1.0% of units were dominated by a single archetype (weight ≥ 0.9; Figure 3B). Accordingly, the soft mixture reconstructed the responses about twice as well as a hard, one-archetype assignment (variance explained 0.72 vs. 0.36), indicating that the reward response is better described as a continuum of mixed temporal profiles rather than as a set of discrete response types.

**Figure 3:**
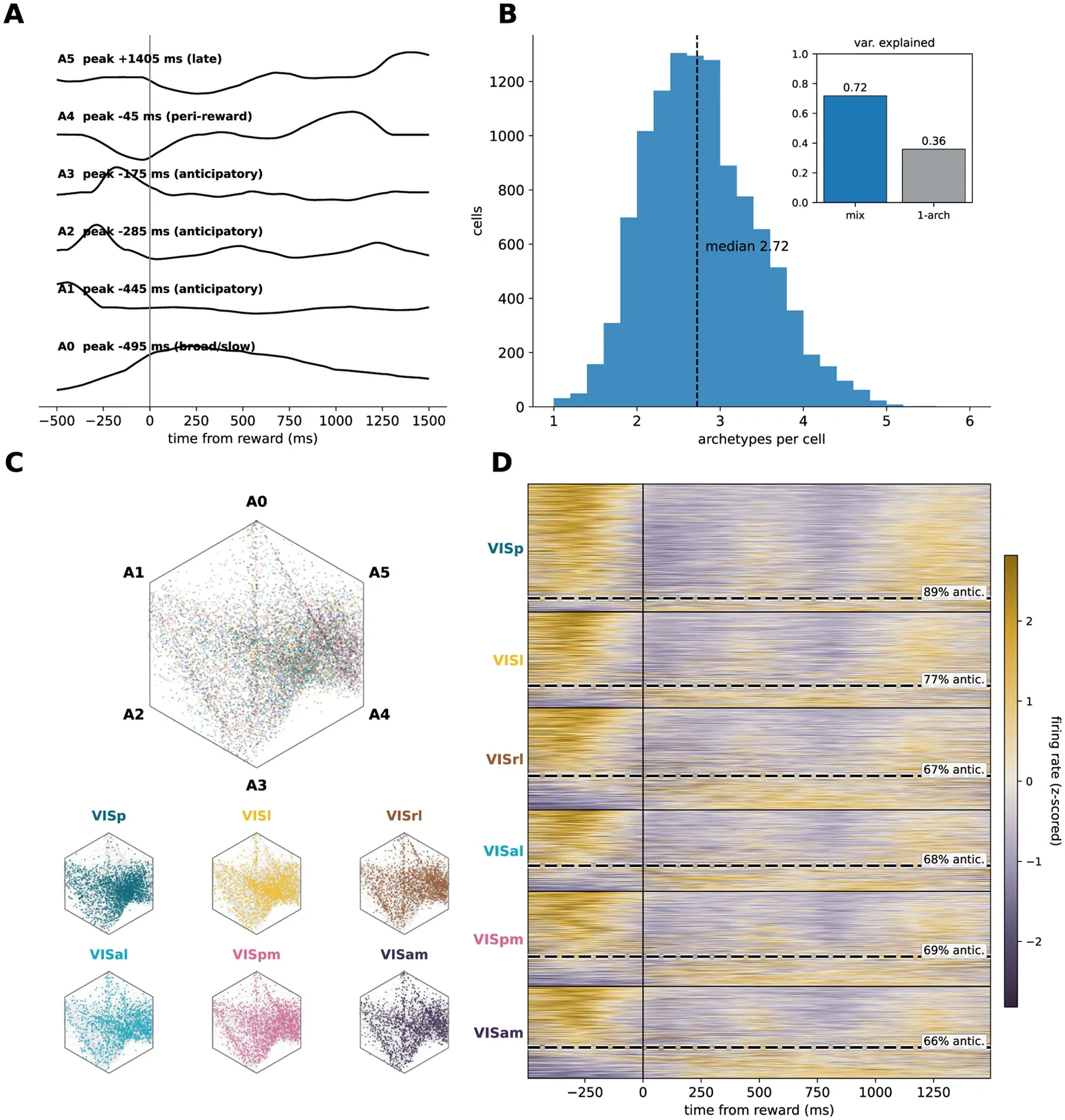
A shared continuum of temporal reward-response profiles. Non-negative matrix factorization (NMF) of the standardized peri-reward PSTHs of the 11,032 cohort units into six signed archetypes (A0–A5; see Methods). **(A)** The six archetype signatures (A0–A5), ordered by peak latency. Each trace is the signed form of an archetype; the label gives its peak time and a short descriptor of its temporal profile: an anticipatory ramp rising before reward (A1–A3), a peri-reward peak (A4), a late peak (A5), or a broad, slow archetype (A0). **(B)** Cells are mixtures rather than discrete types: distribution of the effective number of archetypes per unit (dashed line, median). Inset: variance explained by the soft mixture (mix) vs. a hard, single-archetype assignment (1-arch). **(C)** Repertoire composition by area. Each unit is a point in the six-archetype simplex, whose vertices are the archetypes A0–A5. Top hexagon: all units coloured by area; the six smaller hexagons show each area's distribution alone. The clouds overlap almost entirely. **(D)** Waterfall of peri-reward profiles along the functional visual hierarchy. Each row is one unit of the reward-modulated cohort, and only cohort units are shown; the panel does not include units outside the cohort. Rows give the unit's response (z-score; gold, increase; purple, suppression), grouped by area (VISp to VISam) and sorted within each area by its anticipatory ramp. The dashed line marks the anticipatory/post-reward boundary, labelled with the anticipatory fraction (antic.) of each area.

We next asked whether this continuum is organized by cortical area. Representing each unit by its position in the six-archetype simplex (Figure 3C), the per-area distributions overlapped almost entirely: the between-area variance of the simplex positions was small (η^2^ = 0.004), though reliably above a null obtained by shuffling the area labels (permutation test, *p* < 0.001). The repertoire of temporal reward-response profiles is thus largely shared across the six visual areas, with at most a marginal regional bias in its composition.

Placed along the functional visual hierarchy (Figure 3D), the one regional trend that emerged concerned the balance between anticipatory and peri-reward profiles. Ordering the cohort by area and, within each area, by the sign of its pre- vs. post-reward ramp, the fraction of units with an anticipatory profile was highest in primary visual cortex (VISp, 89%) and in VISl (77%), and lower and similar across the four higher-order areas (66–69%). One possible reading is that anticipatory reward signals are most prevalent in the primary visual cortex and give way to responses locked around reward delivery towards higher-order areas. This trend should be read with caution, however, because suppressed units have, by construction, higher pre- than post-reward activity, the anticipatory-ramp measure partly re-expresses the suppression gradient of Figure 1E, and its ordering depends on the assumed cortical hierarchy. Quantifying that overlap, the two unit-level labels agreed for 80.7% of the cohort (ϕ = 0.56): 91.5% of suppressed units carried an anticipatory ramp, against 39.9% of enhanced units. Standardizing every area to the overall direction composition leaves 31% of the VISp-to-VISam amplitude of the anticipatory-fraction gradient (+0.07 of +0.23), so the gradient is not wholly reducible to the direction label, but is largely accounted for by it. We therefore report it as a descriptive organization of the shared repertoire rather than as an independent regional effect.

## 4 Discussion

We found that reward-related modulation was widespread across the mouse visual cortex but differed substantially across areas, cortical layers, and putative electrophysiological classes. Among 11,032 units that met the bidirectional reward-modulation criteria, decreases in firing were more frequent than increases, accounting for 7,229 units overall. This imbalance was strongest in VISp, where 2,048 of 2,374 reward-modulated units were suppressed, and approached parity in VISam. Within the subset with layer assignments, suppression was most prevalent in layer 4. This detailing extends prior work in two directions. Reward-related activity in the visual cortex has been characterized almost entirely in the primary area (Shuler and Bear, 2006; Chubykin et al., 2013; Poort et al., 2015; Goltstein et al., 2018; Monk et al., 2020), whereas brain-wide and multi-area surveys that span the higher visual areas have treated reward as one of several distributed behavioral variables rather than resolving it in its own right (Steinmetz et al., 2019; International Brain Laboratory, 2025); the primary description of the present dataset likewise decomposed the activity into sensory, change, engagement, novelty and action components without a dedicated reward axis (Bennett et al., 2026). By resolving reward-related modulation jointly across the six visual areas, their cortical layers and their waveform classes, we find that its sign is not spatially uniform along the hierarchy, and that reward-related activity in the visual cortex cannot be summarized as a generalised gain increase.

A notable difference from several studies of reward learning in V1 is the predominant direction of the effect. Conditioning has often been associated with enhanced responses or increased gain for rewarded stimuli and locations (Goltstein et al., 2018; Pakan et al., 2018). In contrast, suppression was the dominant pattern in our reward-aligned population, particularly in VISp. This difference need not represent a direct contradiction. Previous studies frequently quantified responses to reward-predicting visual stimuli or spatial locations, whereas the present analysis was aligned to reward delivery and used a pre-reward interval as a reference. A higher anticipatory baseline, therefore, contributes to an apparently negative reward modulation index, and the size of that contribution is quantified in the Methods: retreating the baseline by 1 s moves the median *M* from −0.1323 to −0.0351 and the fraction of units with *M* < 0 from 71.6% to 54.5%. We retain the adjacent baseline because it is the conventional reference for peri-event indices, but the predominance of negative *M* should accordingly be read as a statement about firing relative to the state immediately preceding reward, and not as evidence of post-reward inhibition. The regional gradient this article reports does not rest on *M*: it derives from the direction label of the specificity index, and its ordering is preserved under every baseline tested. In addition, differences in task structure, training, cortical depth, temporal window, and measurement modality may shift the balance between enhancement and suppression. Our data establish the prevalence and anatomical distribution of the negative modulation, but do not distinguish active inhibition after reward from the termination of anticipatory activity. Reward-evoked suppression is, moreover, not peculiar to the visual cortex. In the nucleus accumbens, a core node of the reward system, appetitive consumption inhibits about half of D1- and D2-type medium spiny neurons, whereas an aversive stimulus predominantly excites them (Domingues et al., 2025). That a decrease in firing is a dominant response to reward, even in canonical reward circuitry, is consistent with our suppression reflecting a genuine reward-related signal, and not only the pre-reward baseline used as reference.

The laminar and waveform results further constrain, but do not identify, the underlying circuit. Layer 4 showed the highest fraction of suppressed units in the layer-assigned cohort. Moreover, narrow-waveform units were more frequently and more strongly suppressed than broad-waveform units. This pattern is not readily explained by a simple model in which neuromodulation or motor corollary discharge recruits fast-spiking interneurons while suppressing excitatory cells. However, extracellular waveform classes are not molecular identities. Narrow waveforms should be described as narrow or putative fast-spiking units, rather than equated with parvalbumin neurons, and the broad class may contain multiple excitatory and inhibitory types. Accordingly, the present findings neither identify a specific interneuron class nor demonstrate that one candidate microcircuit has been selectively engaged.

Cholinergic modulation remains a biologically plausible candidate because it is causally implicated in reward-timing plasticity in V1 (Chubykin et al., 2013) and can suppress different cortical cell classes through more than one circuit configuration. In mouse V1, acetylcholine can facilitate SOM interneurons that inhibit both putative PV cells and pyramidal neurons, providing a qualitative route by which narrow- and broad-waveform populations could decrease together (Chen et al., 2015). This circuit was demonstrated mainly in superficial layers under conditions that did not involve reward, so it cannot be treated as a direct explanation for the layer 4 effect observed here. Recent work also implicates M2 muscarinic receptors in temporal plasticity and reports their enrichment in thalamorecipient layer 4 and in a subset of deep SOM neurons in mouse V1 (Sarkar et al., 2024). An additional, non-exclusive route is top-down input from the orbitofrontal cortex: activating OFC axons in V1 suppresses visual responses through recruitment of local SST interneurons, whereas disrupting this projection impairs visual associative learning (Liu et al., 2020). These anatomical and causal observations strengthen the plausibility of cholinergic and top-down contributions, but our recordings did not measure acetylcholine release, receptor activation, or molecular cell identity. We therefore interpret acetylcholine as a testable hypothesis, not as the demonstrated cause of reward-related suppression.

Motor and arousal signals provide an important alternative or complementary explanation. Reward delivery in the Allen task is closely coupled to licking and may also be accompanied by changes in posture, facial movement, running, and pupil-linked state. Visual cortical neurons can combine reward-related visual information with self-motion signals (Pakan et al., 2018), and corollary discharge can suppress sensory cortical activity through local inhibitory circuits, although the best-resolved synaptic example comes from auditory cortex (Schneider et al., 2014). The active vs. passive comparison used here establishes that the selected responses depend on the behavioral context containing reward and licking, but it does not separate these events because both are absent during passive replay. The two events are, moreover, effectively simultaneous: across the 16,330 earned rewards of the 103 sessions, water was delivered a median of 9.8 ms after the nearest lick (95th percentile 17.3 ms, all within 20 ms), so no choice of alignment separates them at the temporal resolution of the analyses reported here.

We therefore ran the contrast that most directly separates reward from licking, hits vs. false alarms, that is, licking with water against licking without it. It is severely underpowered rather than infeasible, and we report it as such. Only 486 false alarms were available in the 102 sessions covered by this family, a median of four per session against a median of 160 hits, so that a Mann–Whitney comparison at 80% power and α = 0.05 would require a probability of superiority of 0.91, close to complete separation of the two trial distributions; in 26 sessions, no effect size whatever would reach that threshold. Even so, between 14.6% (VISp) and 22.2% (VISpm) of cohort units differed between the two conditions after false-discovery-rate correction, with a median specificity index of −0.56 in VISp, that is, deeper suppression when the lick was rewarded. Because the test is underpowered, the units that did not differ cannot be read as evidence of equivalence.

A better-powered version of the same control is available within the dataset. Unrewarded lick bouts occur about a hundred times more often than false alarms (median 415 per session), and comparing the lick of a hit against the onset of an unrewarded bout, 74.9% (VISp) to 88.8% (VISrl) of cohort units differed after correction, again with deeper suppression on rewarded licks in VISp (median specificity index −0.50). This contrast carries a caveat of its own: the image re-presentations that follow a hit fall inside the post-lick window and do not follow an unrewarded bout, so it isolates residual visual input together with reward rather than reward alone. Neither contrast entered the selection of the cohort. The rapid post-reward component may therefore still contain sensory, consummatory, motor, and neuromodulatory contributions, and this unresolved overlap remains a principal limitation of the current study.

The temporal diversity of the responses suggests that reward-related modulation is not a single event-locked signal. Anticipatory ramps and rapid post-reward transients could reflect different mixtures of expectation, movement preparation, reward consumption, and state change. This interpretation is consistent with the primary Visual Behavior Neuropixels resource, which found that sensory, task, and action variables coexist across the visual system, and that action-related units are present even in early visual cortex (Bennett et al., 2026).

The temporal structure of these responses is organized not as a set of discrete types but as a continuum. Most units combined several temporal archetypes rather than belonging to a single one, and the soft-mixture description reconstructed the responses about twice as well as a single-archetype assignment. This repertoire of profiles was, moreover, largely shared across the six visual areas, with at most a marginal regional bias in its composition. Rather than each area possessing its own type of reward response, the visual cortex appears to express a common basis set of temporal profiles, recruited in similar proportions along the hierarchy. This reading is consistent with the primary description of this dataset, in which sensory, task, and action variables coexist in a distributed, mixed fashion across the visual system (Bennett et al., 2026), and with brain-wide maps showing reward variables represented across widely distributed circuits (International Brain Laboratory, 2025). The one regional trend that emerged, a higher prevalence of anticipatory profiles in primary than in higher-order areas, should be read with caution, because it partly re-expresses the suppression gradient and depends on the assumed cortical hierarchy.

Whether the predominance of suppression generalizes is directly testable in the IBL brain-wide map (International Brain Laboratory, 2025), although the extension is only partly straightforward. Detection would transfer unchanged, since it requires only spike times and reward times, and that release includes the visual cortical recordings among its 75,708 well-isolated neurons. Our hit-vs.-miss contrast also has a counterpart, as IBL trials end either in a water reward or in a noise burst with a time-out. The passive-replay contrast has no direct counterpart. IBL sessions do include a reward-free and task-free visual period, a five-minute receptive-field mapping block run at the end of the session in 504 of 699 insertions, but it presents random sparse-noise stimuli rather than a replay of the task stimuli, so it cannot provide the matched-stimulus comparison that Family A requires. Reward specificity would therefore have to rest on a different control, such as the condition-combined Mann–Whitney test used there to hold the remaining task variables constant. Two further differences would shape any comparison. Reward is earned by a wheel turn and consumed by licking, which the authors note they could not separate from a more abstract representation of reward, and the released analyses do not classify units by waveform, so the narrow/broad split would have to be re-derived rather than transferred.

A complementary interpretive frame for these observations is predictive coding, in which higher cortical areas convey predictions through feedback pathways while lower areas signal the residual error, a scheme originally formulated for the visual cortex (Rao and Ballard, 1999) and later mapped onto the laminar microcircuit (Bastos et al., 2012; Friston, 2010; Keller and Mrsic-Flogel, 2018). In the mouse visual cortex, layer 2/3 neurons carry mismatch signals between predicted and actual visual feedback (Keller et al., 2012), respond to the omission of an expected stimulus (Fiser et al., 2016), and acquire these responses through an experience-dependent, somatostatin-mediated balance of visual and motor input (Attinger et al., 2017). Our data are compatible with, but do not establish, such an account: the anticipatory ramp preceding reward could correspond to a prediction, and the reward-locked modulation to the response evoked when the outcome arrives, with the layer-4 and narrow-spiking involvement echoing the interneuron dependence of cortical mismatch responses. At a larger scale, the presence of reward-related modulation across all six areas, graded along the hierarchy, is consistent with proposals that reward-based computation is distributed and hierarchically organized across the cortex rather than confined to dedicated centers (Hunt and Hayden, 2017; Vickery et al., 2011). We stress that the current dataset cannot separate prediction from movement, nor adjudicate the feedforward and feedback contributions; predictive coding is offered here as a testable interpretation, not a demonstrated mechanism.

Beyond the average reward-aligned response, the modulated cohort differed in the ongoing statistics of population activity across the active block. The summed firing rate of the RW+ cohort operated in a lower-variability regime, with a smaller coefficient of variation than the non-modulated population (RW-), consistently across all six areas. The two populations also differed in how much temporal variability they shared across areas: the co-fluctuation of CV(t) was strongest within the RW- population and weakest within RW+. One reading is that the non-modulated population tracks more closely a globally shared cortical state signal, plausibly linked to locomotion and arousal, whereas the reward-modulated cohort is more area-specific and more decoupled from this common signal. This is consistent with the strong modulation of visual cortical activity by behavioral state and self-motion (Niell and Stryker, 2010; Pakan et al., 2018) and with the shared, non-sensory variability described in this cortex (Denfield et al., 2018). Finally, the increase in variability from the pre- to the post-stimulus window, robust in the non-modulated population in both blocks, was weaker in the modulated cohort and, in the higher-order areas, contingent on the active context, being reduced in passive replay. This context dependence is congruent with the reward-specificity criterion that defined the cohort, whose selected signal requires the behavioral context containing reward and licking. These variability measures should be read with their methodological scope in mind: the CV is not corrected for mean firing rate, matching the unit count does not remove the sensitivity of the correlation to the noise level of the summed series, and RW+ is by construction a population selected by reward modulation, so part of the contrast may reflect that selection or the temporal structure of the active task.

Several limitations define the scope of these conclusions. First, the study is observational and cannot identify the source or causal role of the reward-related signals. Second, reward delivery, licking, and changes in behavioral state are temporally entangled. Third, waveform and CCF-based annotations provide useful anatomical stratification but do not replace molecular identification. Fourth, the selected cohort and its prevalence depend on the statistical null and inclusion criteria; our conclusions concern the spatial, laminar, and temporal organization within the prespecified bidirectional ISI cohort, rather than the absolute fraction of all visual cortical neurons that encode reward. Future experiments should separate reward from licking and visual change, and combine cell-type-specific measurements with direct sensors of acetylcholine and catecholamines, and manipulate candidate basal-forebrain and top-down pathways. Dopamine and noradrenaline remain possible contributors, but the present data and the limited functional literature in mouse V1 do not support assigning the observed suppression to either system. Overall, our results position the visual cortex as a heterogeneous component of distributed reward-related processing, with a particularly strong suppression-dominant regime in VISp that provides a concrete target for causal circuit experiments.

## Data Availability

Publicly available datasets were analyzed in this study. The Allen Visual Behavior Neuropixels dataset can be found in the DANDI Archive, dandiset 000713 (https://doi.org/10.48324/dandi.000713/0.240702.1725). The analysis code is available from the corresponding authors upon reasonable request.

## Supplementary material

Supplementary Figure S1 Rank selection for the non-negative matrix factorization: variance explained, marginal gain, cross-fit archetype stability, and the continuity of the representation for ranks 2 to 8.
