# Supplementary material for: Reward coding across mouse visual cortical areas
Source: Front Behav Neurosci. 2026 Oct 28;20:1944695. doi: 10.3389/fnbeh.2026.1944695 (PMC13752079; doi:10.3389/fnbeh.2026.1944695)
Supplement: Supplementary file 1 [file Data_Sheet_1.pdf]

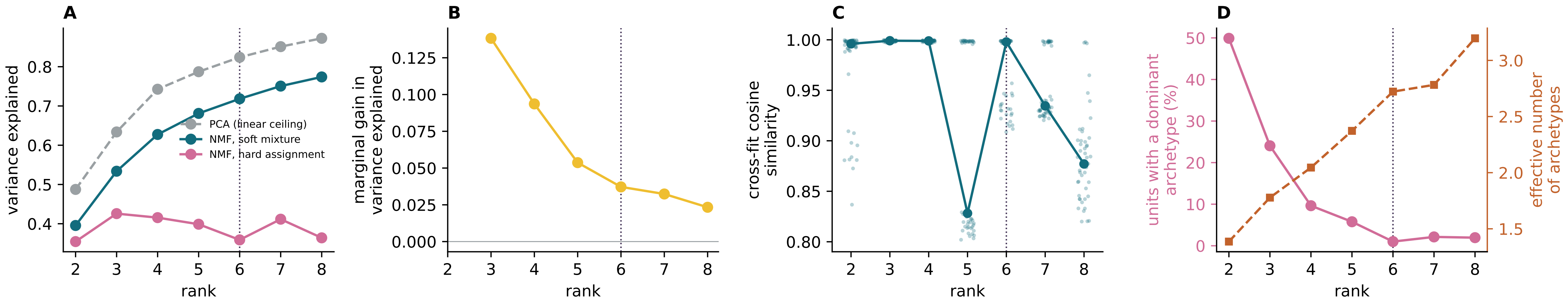

Supplementary Figure S1. Rank selection for the non-negative matrix factorization. All panels use the same response matrix as the published decomposition (11,032 cohort units × 200 bins of the active block) and the same settings (two-channel representation, nndsvda initialization, coordinate-descent solver, tol 10^{-4}, 600 iterations). The dotted line marks rank 6, the rank used in the paper. (A) Variance explained against rank for the soft mixture, for the hard assignment in which each unit keeps only its dominant archetype, and for a PCA with the same number of components, shown as a linear ceiling. The soft mixture rises steadily while the hard assignment does not improve with rank, so added archetypes describe graded mixtures rather than further discrete types. (B) Marginal gain in variance explained from one rank to the next. It decreases smoothly and never saturates, so reconstruction error provides no elbow and does not by itself select a rank. (C) Cross-fit stability: disjoint random halves of the units were factorized independently and their archetypes matched by cosine similarity. Dots, the 50 random partitions, jittered horizontally; line, the median. The distribution is bimodal: a partition either recovers the same archetypes (cosine above 0.99) or settles on a different solution, so a mean over partitions is not a typical value. Ranks 3 and 4 reproduce in every partition, and beyond them reproduction becomes intermittent: rank 6 reproduces in 56% of partitions, against 48% at rank 5, 30% at rank 7 and 6% at rank 8. (D) Continuity of the representation against rank: fraction of units whose dominant archetype carries at least 90% of the normalized weight (left axis), and median effective number of archetypes per unit, the exponential of the entropy of its normalized weights (right axis). Units become less, not more, dominated by a single archetype as the rank grows, so the continuum reported in the main text is not a consequence of the chosen rank.
